# Supplementary material for: Prevalence Trend and Disparities in Rheumatoid Arthritis among US Adults, 2005–2018
Source: J Clin Med. 2021 Jul 26;10(15):3289. doi: 10.3390/jcm10153289 (PMC8348893; doi:10.3390/jcm10153289)
Supplement: Supplementary file 1 [file jcm-10-03289-s001.zip › jcm-1279949-supplementary.pdf]

Supplementary Table S1. Risk factors related to rheumatoid arthritis for men and women of the study sample in 7 National Health and Nutrition Examination Surveys, from 2005 to 2018

	2005-2006 (n=4,459)	2007-2008 (n=5,084)	2009-2010 (n=5,399)	2011-2012 (n=4,801)	2013-2014 (n=5,094)	2015-2016 (n=4,846)	2017-2018 (n=4,488)
Men, unweighted No. (Weighted %)							
Obesity	680 (33.00)	841(32.22)	938 (36.00)	757 (33.56)	806 (35.15)	847 (38.62)	865 (43.32)
Current smoker	560 (27.50)	647 (25.94)	628 (21.90)	599 (23.61)	561 (21.06)	530 (20.09)	477 (19.46)
Physical inactive	170 (6.13)	564 (14.56)	604 (17.48)	469 (16.76)	496 (18.72)	493 (15.96)	449 (15.67)
Women, unweighted No. (Weighted %)							
Obesity	901 (35.90)	1020 (35.02)	1140 (36.49)	980 (36.60)	1122 (40.68)	1122 (41.43)	1024 (42.97)
Current smoker	413 (20.49)	503 (19.90)	551 (19.09)	369 (16.03)	491 (19.32)	381 (15.94)	340 (15.53)
Physical inactive	274 (10.06)	899 (27.69)	895 (28.26)	717 (24.89)	811 (28.65)	776 (25.20)	692 (25.00)

Supplementary Figure S1. Age-adjusted prevalence of rheumatoid arthritis by weight status in different race groups, 2005-2006 through 2017-2018 Figure (a) shows the prevalence of Non-Hispanic Caucasian by weight status; Figure (b) shows the prevalence of Hispanic by race by weight status; Figure (c) shows the prevalence of Non-Hispanic African American by race by weight status

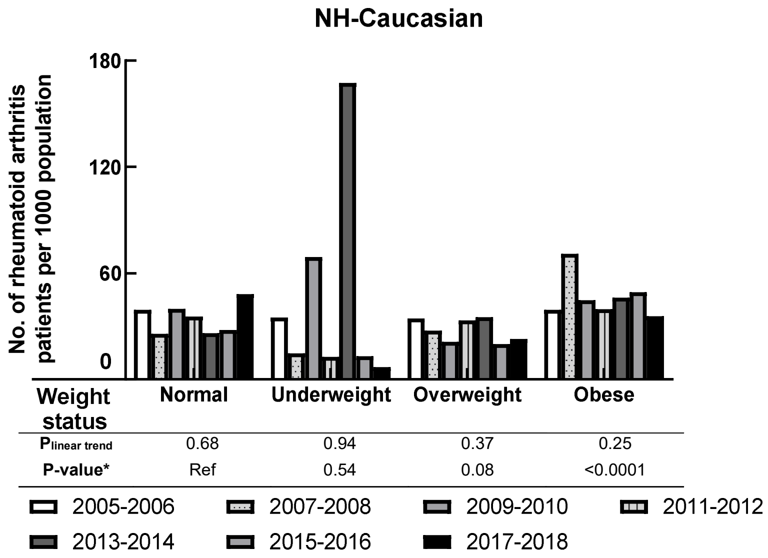

(a)

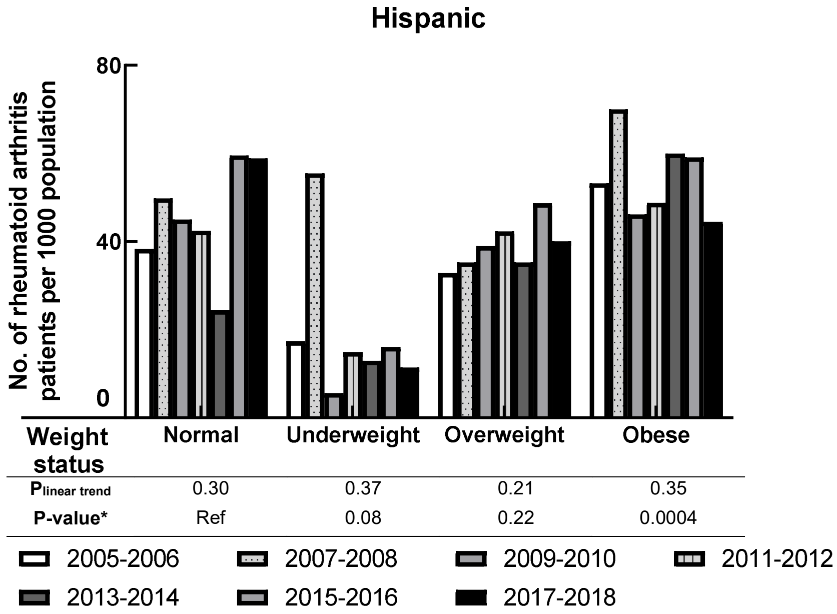

(b)

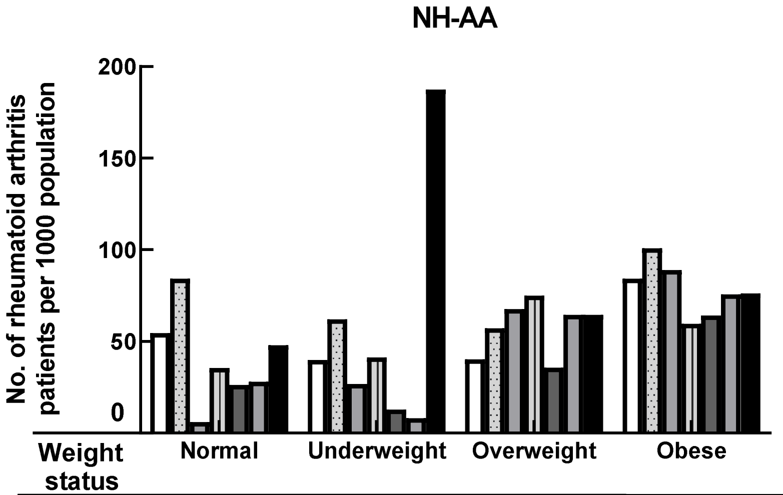

	Normal	Underweight	Overweight	Obese
$P_{linear\ trend}$	0.07	0.29	0.41	0.17
P-value*	Ref	0.91	0.73	0.002

2005-2006 2007-2008 2009-2010 2011-2012
2013-2014 2015-2016 2017-2018

(c)
